# 3-(4-Iodo­phen­yl)-2,3-di­hydro-1*H*-benzo[*f*]chromen-1-one

**DOI:** 10.1107/S2414314620001108

**Published:** 2020-01-31

**Authors:** Raven Dean, Chelsea N. Miller, Sarah K. Zingales, Clifford W. Padgett

**Affiliations:** a Georgia Southern University, 11935 Abercorn St, Department of Chemistry and Biochemistry, Savannah GA 31419, USA; University of Aberdeen, Scotland

**Keywords:** crystal structure, naphtho­pyran, flavone, halogen bond

## Abstract

In the title compound, C_19_H_13_IO_2_, the dihedral angle between the naphthyl ring system and the pendant iodo­phenyl ring is 72.48 (11)°. In the crystal, C—H⋯π inter­actions and I⋯O [3.293 (2) Å] halogen bonds are observed, which combine to generate a herringbone packing motif.

## Abstract 2

In the crystal of the title flavone derivative, O⋯I halogen bonding and T–shaped π–stacking combine to generate a herringbone packing motif.

## Graphical abstract

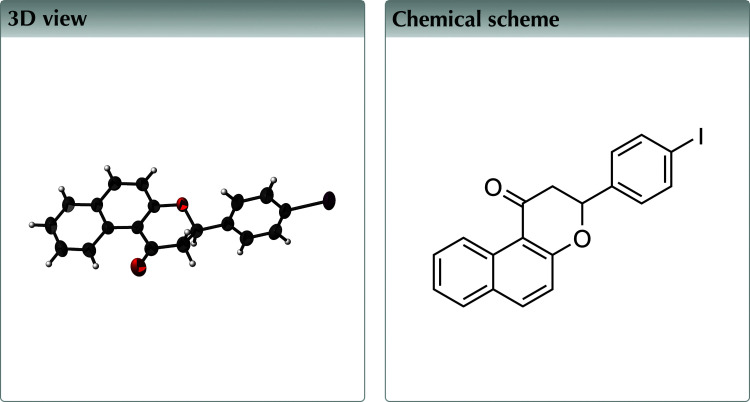

## Structure description

Traditional CORMS (carbon monoxide-releasing mol­ecules) contain metal carbonyls whereas photoCORMS have recently become of inter­est because of their ability to release CO in biological systems. Our group is particularly inter­ested in the extended flavonol motif as it has been shown to release CO qu­anti­tatively with visible light (Popova *et al.*, 2017 ▸). Typically, we synthesize these flavonols in two steps from an acetyl naphthol and an aromatic aldehyde. The first step is an aldol condensation, followed by oxidative cyclization. However, if no oxidant is added, the 2′–hy­droxy­chalcone inter­mediate can cyclize to a flavanone under basic conditions (Furlong *et al.*, 1985 ▸). In our quest to synthesize a novel flavonol (2-hy­droxy-3-(4-iodo­phen­yl)-1*H*-naphtho­[2,1-*b*]pyran-1-one), we serendipitously synthesized the title flavanone.

**Figure 1:**
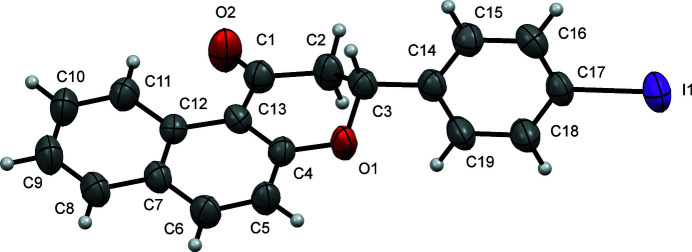
A view of the mol­ecular structure of the title compound, showing the atom labeling. Displacement ellipsoids are drawn at the 50% probability level.

**Figure 2:**
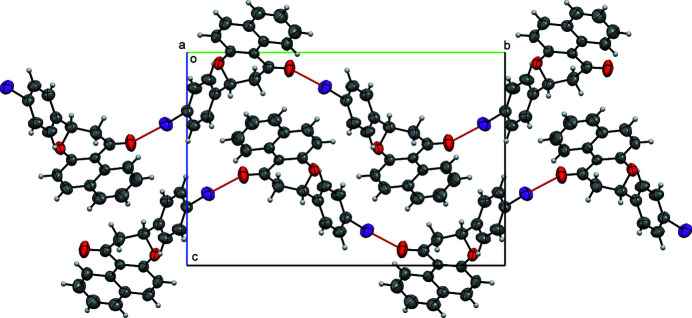
Crystal packing diagram of the title compound, viewed along the *a* axis. O⋯I halogen bonds are indicated has red lines.

In the title mol­ecule (Fig. 1 ▸), the iodo­phenyl ring is tilted by 72.48 (11)° with respect to the naphthyl ring system. No hydrogen bonding is observed in the extended structure. T-shaped π-stacking with *Cg*1⋯*Cg*2^i^ = 4.929 (2) Å [symmetry code: (i) 1 − *x*, 1 − *y*, 1 − *z*] and C6—H6⋯*Cg*2^i^ = 154.5 (3)°, where *Cg*1 is the centroid of the pyran­one ring containing atoms C4–C7/C12/C13 and *Cg*2 is the centroid of the iodo­phenyl ring containing atoms C14–C19 (Burley & Petsko, 1985 ▸). I⋯O halogen bonds between neighboring mol­ecules form a chain that runs parallel to the *b-*axis direction. The I1⋯O2^ii^ distance is 3.293 (2) Å, with C17—I1⋯O2^ii^ and I1⋯O2^ii^—C1^ii^ angles of 177.21 (10) and 127.9 (2)°, respectively [symmetry code: (ii) − 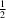 − *x*, − 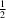 + *y*, 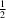 − *z*]. This I⋯O separation is some 0.25 Å shorter than van der Waals’inter­action distance of 3.5 Å (Rissanen, 2008 ▸) The crystal structure exhibits a herringbone pattern (Fig. 2 ▸) with mol­ecules linked into [010] chains by the halogen bonding; neighboring layers are held together with van der Waals inter­actions along with T-shaped π-stacking.

## Synthesis and crystallization

1-Acetyl-2-naphthol (164 mg, 0.88 mmol) and 4-iodo­benzaldehyde (205 mg, 0.88 mmol) were dissolved in ethanol (5 ml). An NaOH solution (5 *M*, 0.76 ml) was added and the reaction was stirred until a precipitate formed. The reaction mixture was acidified to pH 4 with glacial acetic acid. The solids were filtered and taken directly to the next step. (*E*)-1-(2-Hy­droxy­naphthalen-1-yl)-3-(4-iodo­phen­yl)prop-2-en-1-one was then suspended in ethanol (10 ml). An NaOH solution (5 *M*, 0.12 ml) was added and the reaction stirred until a precipitate formed. The reaction mixture was acidified to pH 1 with HCl (6 *M*). The white solid was collected by filtration and slow evaporation of a solution of the title compound in ethyl acetate gave colorless crystals (108 mg, 30% yield over two steps).

^1^H NMR (300 MHz, (CDCl_2_) δ = 9.46 (*d*, *J* = 8.6 Hz, 1H), 7.95 (*d*, *J* = 8.9 Hz, 1H), 7.80–7.75 (*m*, 3H), 7.65 (*t*, *J* = 7.9 Hz, 1H), 7.44 (*t*, *J* = 7.6 Hz, 1H), 7.26 (*d*, *J* = 8.6 Hz, 2H), 7.16 (*d*, *J* = 8.9 Hz, 1H), 5.54 (*dd*, *J* = 13.4, 3.1 Hz, 1H), 3.16 (*dd*, *J* = 16.5, 13.2 Hz, 1H), 2.95 (*dd*, *J* = 16.5, 3.0 Hz, 1H) ppm.

## Refinement

Crystal data, data collection and structure refinement details are summarized in Table 1 ▸.

## Supplementary Material

Crystal structure: contains datablock(s) I. DOI: 10.1107/S2414314620001108/hb4334sup1.cif

Structure factors: contains datablock(s) I. DOI: 10.1107/S2414314620001108/hb4334Isup2.hkl

Supporting information file. DOI: 10.1107/S2414314620001108/hb4334Isup3.cml

CCDC reference: 1980111

Additional supporting information:  crystallographic information; 3D view; checkCIF report

## Figures and Tables

**Table 1:** Experimental details

Crystal data	
Chemical formula	C_19_H_13_IO_2_
*M*_r_	400.19
Crystal system, space group	Monoclinic, *P*2_1_/*n*
Temperature (K)	170
*a*, *b*, *c* (Å)	7.0481 (3), 18.2185 (8), 12.6391 (6)
β (°)	104.947 (4)
*V* (Å^3^)	1568.02 (12)
*Z*	4
Radiation type	Mo *K*α
μ (mm^−1^)	2.05
Crystal size (mm)	0.77 × 0.34 × 0.34

Data collection	
Diffractometer	Rigaku XtaLAB mini
Absorption correction	Multi-scan (*CrysAlis PRO*; Rigaku OD, 2018 ▸)
*T*_min_, *T*_max_	0.738, 1.000
No. of measured, independent and observed [*I* > 2σ(*I*)] reflections	23424, 5656, 3493
*R*_int_	0.035
(sin θ/λ)_max_ (Å^−1^)	0.768

Refinement	
*R*[*F* ^2^ > 2σ(*F* ^2^)], *wR*(*F* ^2^), *S*	0.045, 0.096, 1.12
No. of reflections	5656
No. of parameters	199
H-atom treatment	H-atom parameters constrained
Δρ_max_, Δρ_min_ (e Å^−3^)	0.72, −0.81

Computer programs: *CrysAlis PRO* (Rigaku OD, 2018 ▸), *SHELXT* (Sheldrick, 2015*a* ▸), *SHELXL* (Sheldrick, 2015*b* ▸) and *OLEX2* (Dolomanov *et al.*, 2009 ▸).

## full crystallographic data

### Crystal data

**Table:**

C_19_H_13_IO_2_	*F*(000) = 784
*M**_r_* = 400.19	*D*_x_ = 1.695 Mg m^−^^3^
Monoclinic, *P*2_1_/*n*	Mo *K*α radiation, λ = 0.71073 Å
*a* = 7.0481 (3) Å	Cell parameters from 5447 reflections
*b* = 18.2185 (8) Å	θ = 2.0–28.8°
*c* = 12.6391 (6) Å	µ = 2.05 mm^−^^1^
β = 104.947 (4)°	*T* = 170 K
*V* = 1568.02 (12) Å^3^	Prism, colorless
*Z* = 4	0.77 × 0.34 × 0.34 mm

### Data collection

**Table:**

Rigaku XtaLAB mini diffractometer	3493 reflections with *I* > 2σ(*I*)
Detector resolution: 13.6612 pixels mm^-1^	*R*_int_ = 0.035
profile data from ω–scans	θ_max_ = 33.1°, θ_min_ = 2.0°
Absorption correction: multi-scan (CrysAlis PRO; Rigaku OD, 2018)	*h* = −10→10
*T*_min_ = 0.738, *T*_max_ = 1.000	*k* = −27→25
23424 measured reflections	*l* = −19→18
5656 independent reflections	

### Refinement

**Table:**

Refinement on *F*^2^	0 restraints
Least-squares matrix: full	Hydrogen site location: inferred from neighbouring sites
*R*[*F*^2^ > 2σ(*F*^2^)] = 0.045	H-atom parameters constrained
*wR*(*F*^2^) = 0.096	*w* = 1/[σ^2^(*F*_o_^2^) + (0.0197*P*)^2^ + 1.6677*P*] where *P* = (*F*_o_^2^ + 2*F*_c_^2^)/3
*S* = 1.12	(Δ/σ)_max_ = 0.001
5656 reflections	Δρ_max_ = 0.72 e Å^−^^3^
199 parameters	Δρ_min_ = −0.81 e Å^−^^3^

### Special details

**Table:**

Geometry. All esds (except the esd in the dihedral angle between two l.s. planes) are estimated using the full covariance matrix. The cell esds are taken into account individually in the estimation of esds in distances, angles and torsion angles; correlations between esds in cell parameters are only used when they are defined by crystal symmetry. An approximate (isotropic) treatment of cell esds is used for estimating esds involving l.s. planes.
Refinement. All C–bound H atoms were positioned geometrically and refined as riding, with C—H = 0.93 or 0.96 Å and *U*_iso_(H) = 1.2*U*_eq_(C) or *U*_iso_(H) = 1.5*U*_eq_(C) for C(H) and CH_3_ groups, respectively.

### Fractional atomic coordinates and isotropic or equivalent isotropic displacement parameters (Å^2^)

**Table:**

	*x*	*y*	*z*	*U*_iso_*/*U*_eq_	
I1	−0.50948 (3)	0.43392 (2)	0.16336 (2)	0.06235 (10)	
O1	0.3551 (3)	0.60041 (12)	0.4492 (2)	0.0521 (6)	
C1	0.3838 (5)	0.75621 (18)	0.4302 (3)	0.0500 (8)	
O2	0.3855 (4)	0.82269 (13)	0.4246 (3)	0.0732 (8)	
C2	0.2006 (5)	0.71359 (18)	0.3764 (3)	0.0579 (9)	
H2A	0.111796	0.713332	0.425697	0.069*	
H2B	0.131632	0.738742	0.307874	0.069*	
C3	0.2426 (4)	0.63579 (18)	0.3505 (3)	0.0482 (8)	
H3	0.323461	0.636549	0.296041	0.058*	
C4	0.5196 (4)	0.63685 (16)	0.5048 (3)	0.0418 (7)	
C5	0.6575 (5)	0.59171 (18)	0.5759 (3)	0.0522 (8)	
H5	0.631204	0.540920	0.581550	0.063*	
C6	0.8277 (5)	0.6209 (2)	0.6361 (3)	0.0556 (9)	
H6	0.916930	0.590867	0.687195	0.067*	
C7	0.8749 (4)	0.69535 (18)	0.6244 (3)	0.0471 (7)	
C8	1.0575 (5)	0.7242 (2)	0.6837 (3)	0.0610 (10)	
H8	1.146842	0.693508	0.733673	0.073*	
C9	1.1078 (6)	0.7950 (2)	0.6704 (4)	0.0708 (11)	
H9	1.231105	0.813773	0.710556	0.085*	
C10	0.9759 (6)	0.8396 (2)	0.5970 (4)	0.0716 (12)	
H10	1.012377	0.888683	0.586249	0.086*	
C11	0.7944 (5)	0.81461 (19)	0.5397 (3)	0.0559 (9)	
H11	0.706231	0.846849	0.491926	0.067*	
C12	0.7382 (4)	0.74111 (17)	0.5516 (3)	0.0423 (7)	
C13	0.5495 (4)	0.71092 (16)	0.4953 (2)	0.0387 (6)	
C14	0.0648 (4)	0.58838 (18)	0.3059 (3)	0.0474 (7)	
C15	0.0197 (5)	0.56413 (19)	0.1993 (3)	0.0537 (8)	
H15	0.101446	0.577554	0.153307	0.064*	
C16	−0.1444 (5)	0.5201 (2)	0.1580 (3)	0.0546 (8)	
H16	−0.174482	0.503502	0.084224	0.066*	
C17	−0.2617 (4)	0.50090 (18)	0.2245 (3)	0.0488 (8)	
C18	−0.2188 (5)	0.5240 (2)	0.3317 (3)	0.0591 (9)	
H18	−0.299918	0.509947	0.377662	0.071*	
C19	−0.0551 (5)	0.5683 (2)	0.3719 (3)	0.0592 (9)	
H19	−0.025515	0.584942	0.445616	0.071*	

### Atomic displacement parameters (Å^2^)

**Table:**

	*U*^11^	*U*^22^	*U*^33^	*U*^12^	*U*^13^	*U*^23^
I1	0.04458 (13)	0.06445 (16)	0.06874 (17)	−0.00698 (11)	−0.00216 (10)	−0.01116 (13)
O1	0.0414 (11)	0.0401 (11)	0.0626 (15)	−0.0048 (9)	−0.0087 (10)	0.0070 (10)
C1	0.0424 (16)	0.0458 (18)	0.056 (2)	0.0027 (14)	0.0030 (14)	0.0040 (15)
O2	0.0588 (15)	0.0414 (13)	0.103 (2)	0.0045 (11)	−0.0094 (14)	0.0091 (14)
C2	0.0417 (16)	0.0484 (19)	0.072 (2)	0.0019 (14)	−0.0065 (16)	0.0077 (17)
C3	0.0384 (15)	0.0525 (19)	0.0481 (19)	−0.0004 (14)	0.0010 (13)	0.0027 (14)
C4	0.0364 (14)	0.0397 (15)	0.0469 (17)	−0.0030 (12)	0.0065 (12)	0.0027 (13)
C5	0.0466 (17)	0.0386 (16)	0.062 (2)	−0.0029 (13)	−0.0030 (15)	0.0093 (15)
C6	0.0420 (16)	0.054 (2)	0.061 (2)	0.0007 (15)	−0.0045 (15)	0.0114 (17)
C7	0.0366 (14)	0.0510 (18)	0.0487 (18)	−0.0045 (13)	0.0020 (13)	−0.0002 (14)
C8	0.0463 (18)	0.068 (2)	0.059 (2)	−0.0095 (17)	−0.0036 (16)	0.0030 (18)
C9	0.055 (2)	0.073 (3)	0.073 (3)	−0.023 (2)	−0.0036 (19)	−0.002 (2)
C10	0.068 (2)	0.063 (2)	0.073 (3)	−0.028 (2)	0.000 (2)	0.001 (2)
C11	0.0565 (19)	0.0462 (18)	0.060 (2)	−0.0087 (15)	0.0055 (16)	0.0028 (16)
C12	0.0408 (15)	0.0427 (16)	0.0421 (16)	−0.0056 (13)	0.0083 (12)	−0.0004 (13)
C13	0.0375 (14)	0.0400 (15)	0.0358 (15)	0.0013 (12)	0.0042 (11)	−0.0036 (12)
C14	0.0369 (15)	0.0473 (17)	0.0529 (19)	−0.0003 (13)	0.0023 (13)	0.0029 (14)
C15	0.0447 (17)	0.060 (2)	0.054 (2)	−0.0026 (16)	0.0096 (14)	−0.0012 (17)
C16	0.0472 (17)	0.064 (2)	0.0464 (19)	0.0016 (16)	0.0003 (14)	−0.0047 (16)
C17	0.0377 (15)	0.0493 (18)	0.0514 (19)	0.0005 (13)	−0.0027 (13)	−0.0035 (15)
C18	0.0506 (19)	0.075 (2)	0.049 (2)	−0.0139 (18)	0.0083 (15)	−0.0055 (18)
C19	0.0509 (19)	0.075 (2)	0.0453 (19)	−0.0140 (18)	0.0012 (15)	−0.0091 (18)

### Geometric parameters (Å, º)

**Table:**

I1—C17	2.107 (3)	C8—H8	0.9500
O1—C3	1.446 (4)	C8—C9	1.360 (5)
O1—C4	1.364 (3)	C9—H9	0.9500
C1—O2	1.213 (4)	C9—C10	1.393 (6)
C1—C2	1.510 (4)	C10—H10	0.9500
C1—C13	1.492 (4)	C10—C11	1.375 (5)
C2—H2A	0.9900	C11—H11	0.9500
C2—H2B	0.9900	C11—C12	1.415 (4)
C2—C3	1.502 (5)	C12—C13	1.445 (4)
C3—H3	1.0000	C14—C15	1.374 (5)
C3—C14	1.507 (4)	C14—C19	1.381 (5)
C4—C5	1.406 (4)	C15—H15	0.9500
C4—C13	1.376 (4)	C15—C16	1.394 (5)
C5—H5	0.9500	C16—H16	0.9500
C5—C6	1.353 (4)	C16—C17	1.368 (5)
C6—H6	0.9500	C17—C18	1.376 (5)
C6—C7	1.413 (5)	C18—H18	0.9500
C7—C8	1.413 (4)	C18—C19	1.392 (5)
C7—C12	1.419 (4)	C19—H19	0.9500
			
C4—O1—C3	115.6 (2)	C8—C9—C10	119.0 (3)
O2—C1—C2	120.5 (3)	C10—C9—H9	120.5
O2—C1—C13	124.5 (3)	C9—C10—H10	119.1
C13—C1—C2	114.9 (3)	C11—C10—C9	121.9 (4)
C1—C2—H2A	109.0	C11—C10—H10	119.1
C1—C2—H2B	109.0	C10—C11—H11	119.8
H2A—C2—H2B	107.8	C10—C11—C12	120.3 (3)
C3—C2—C1	112.9 (3)	C12—C11—H11	119.8
C3—C2—H2A	109.0	C7—C12—C13	118.7 (3)
C3—C2—H2B	109.0	C11—C12—C7	117.6 (3)
O1—C3—C2	109.1 (3)	C11—C12—C13	123.7 (3)
O1—C3—H3	108.5	C4—C13—C1	118.4 (3)
O1—C3—C14	106.6 (3)	C4—C13—C12	118.2 (3)
C2—C3—H3	108.5	C12—C13—C1	123.4 (3)
C2—C3—C14	115.5 (3)	C15—C14—C3	120.7 (3)
C14—C3—H3	108.5	C15—C14—C19	119.0 (3)
O1—C4—C5	113.6 (3)	C19—C14—C3	120.3 (3)
O1—C4—C13	124.2 (3)	C14—C15—H15	119.6
C13—C4—C5	122.2 (3)	C14—C15—C16	120.8 (3)
C4—C5—H5	120.1	C16—C15—H15	119.6
C6—C5—C4	119.7 (3)	C15—C16—H16	120.3
C6—C5—H5	120.1	C17—C16—C15	119.4 (3)
C5—C6—H6	119.4	C17—C16—H16	120.3
C5—C6—C7	121.1 (3)	C16—C17—I1	119.9 (2)
C7—C6—H6	119.4	C16—C17—C18	120.8 (3)
C6—C7—C12	119.5 (3)	C18—C17—I1	119.3 (3)
C8—C7—C6	120.5 (3)	C17—C18—H18	120.4
C8—C7—C12	119.9 (3)	C17—C18—C19	119.2 (3)
C7—C8—H8	119.4	C19—C18—H18	120.4
C9—C8—C7	121.2 (3)	C14—C19—C18	120.7 (3)
C9—C8—H8	119.4	C14—C19—H19	119.6
C8—C9—H9	120.5	C18—C19—H19	119.6
